# Acute Calcium Pyrophosphate Crystal Arthritis Occurring Many Years After Total Knee Arthroplasty: A Case Report

**DOI:** 10.7759/cureus.104367

**Published:** 2026-02-27

**Authors:** Hiroaki Matsubara, Hiroshi Kobata, Tsuneaki Kenzaka

**Affiliations:** 1 Department of Internal Medicine, Hyogo Prefectural Tamba Medical Center, Tamba, JPN; 2 Division of Community Medicine and Career Development, Kobe University Graduate School of Medicine, Kobe, JPN

**Keywords:** acute calcium pyrophosphate crystal arthritis, calcium pyrophosphate deposition disease, case report, prosthetic joint infection, pseudogout, total knee arthroplasty

## Abstract

Acute calcium pyrophosphate (CPP) crystal arthritis, also known as pseudogout, is a form of crystal-induced arthritis. It is caused by acute inflammation triggered by CPP crystals deposited in articular cartilage and surrounding intra-articular tissues. A 94-year-old Japanese woman presented with left knee pain. She had undergone bilateral total knee arthroplasty (TKA) at 80 years of age. Septic arthritis of the prosthetic joint was initially suspected. Synovial fluid analysis showed negative Gram staining and no bacterial growth on culture. However, microscopic examination of the synovial fluid revealed CPP dihydrate (CPPD) crystals, leading to a diagnosis of acute CPP crystal arthritis. Celecoxib (200 mg/day) was initiated after admission, resulting in rapid clinical improvement. It has been suggested that residual synovial tissue after TKA may undergo chondrogenic differentiation and produce CPPD crystals, thereby inducing arthritis. Herein, we report a rare case of acute CPP crystal arthritis occurring many years after TKA. Prompt joint aspiration and synovial fluid evaluation are essential to differentiate this condition from septic arthritis of the prosthetic knee.

## Introduction

Calcium pyrophosphate (CPP) crystal deposition is characterized by the accumulation of CPP dihydrate (CPPD) crystals in the articular cartilage and surrounding tissues. Acute CPP crystal arthritis, also known as pseudogout, is a crystal-induced arthritis caused by acute inflammation triggered by CPP crystals deposited in the articular cartilage and periarticular tissues [1]. Primary symptoms include fever and joint pain. The knee is the most commonly affected joint, accounting for 50-88% of cases, followed by the wrist (approximately 20%). Other sites include the ankle, hip, pubic symphysis, shoulders, and spinal ligaments [2]. After total knee arthroplasty (TKA), the articular cartilage is absent, creating a condition wherein CPPD or acute CPP crystal arthritis is generally thought not to occur [3]. However, recent reports have demonstrated that crystal-induced arthritis can still arise in prosthetic joints, likely due to chondrogenic metaplasia of residual synovial tissue, which may continue to generate CPP crystals even after arthroplasty [4,5]. Although these cases remain rare, they highlight that acute CPP crystal arthritis should be considered in the differential diagnosis of acute postoperative knee inflammation.

Importantly, most previously reported cases occurred relatively soon after arthroplasty [4,5], whereas the present case developed more than a decade after TKA, representing an unusually long latency period. This prolonged interval underscores the clinical importance of recognizing that acute CPP crystal arthritis can occur even many years after TKA. Herein, we report a case of acute CPP crystal arthritis that developed many years after TKA.

## Case presentation

A 94-year-old Japanese woman presented with left knee pain. Redness, swelling, and pain in the left knee began two days before presentation and progressively worsened. Her medical history included bilateral TKA at 80 years of age. She had no history of gout. Comorbidities included hypertension, dyslipidemia, and osteoporosis. Her medications included amlodipine (5 mg/day), atorvastatin (5 mg/day), and denosumab, subcutaneously administered every six months.

**Figure 1:**
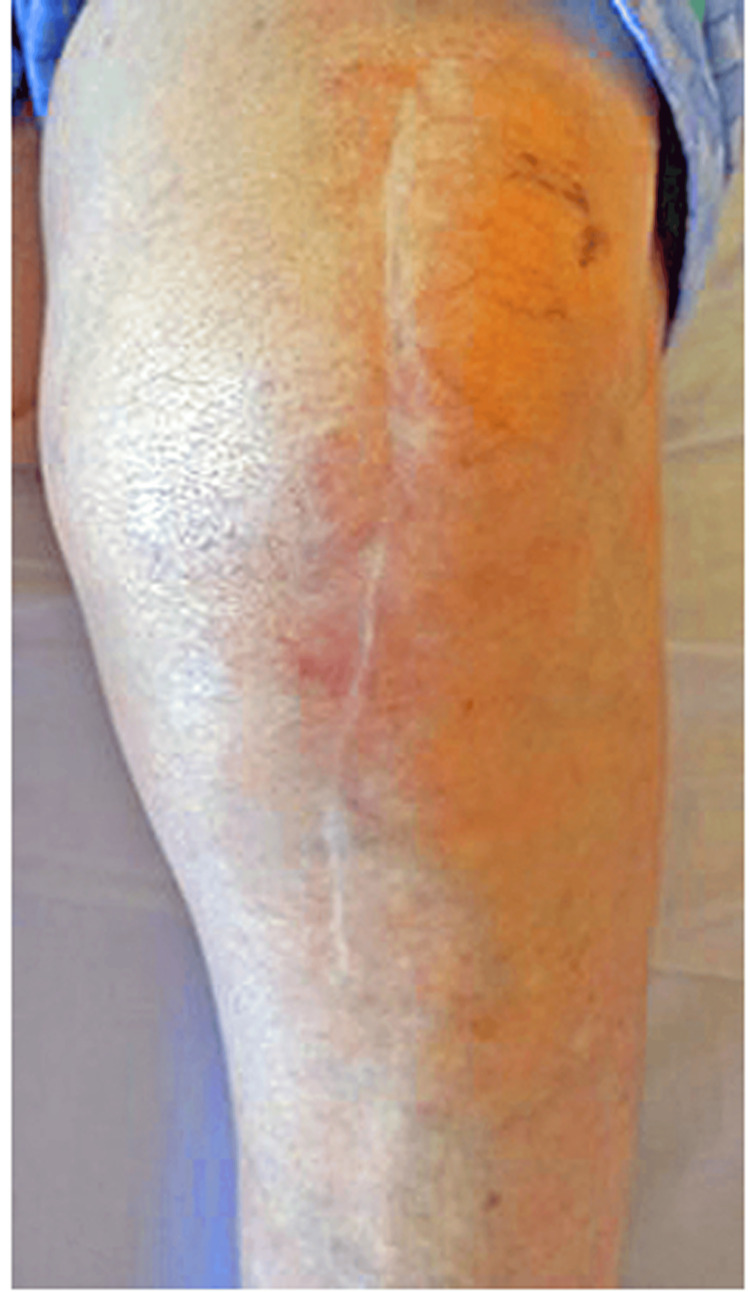
Photo of the left knee The left knee exhibits erythema, swelling, and pain with both active and passive motion, along with patellar ballottement.

On presentation, vital signs were as follows: Glasgow Coma Scale score of E4V5M6, temperature of 37.5°C, oxygen saturation of 93% (room air), blood pressure of 130/76 mmHg, and pulse of 74 beats/min (regular). Physical examination revealed no conjunctival pallor, normal heart or breath sounds, and a soft, non-tender abdomen. The left knee exhibited erythema, swelling, and pain with both active and passive motion, along with patellar ballottement (Figure 1). None of the other joints was affected.

The laboratory findings are listed in Table 1.

**Table 1:** Laboratory data upon admission

Parameter	Recorded value	Standard value
White blood cell count	10,260/µL	4,500-7,500/µL
Neutrophils	78.4%	42-74%
Lymphocytes	14.1%	18-50%
Hemoglobin	12.8 g/dL	11.3-15.2 g/dL
Platelet count	22.5×10^4^/µL	13-35×10^4^/µL
Prothrombin time/International normalized ratio	1.16	0.80-1.20
Activated partial thromboplastin time	32.0 s	26.9-38.1 s
D-dimer	5.6 μg/mL	<1.0 μg/mL
C-reactive protein	15.82 mg/L	≤0.60 mg/dL
Procalcitonin	0.50 ng/mL	≤0.05 ng/mL
Total protein	7.3 g/dL	6.9-8.4 g/dL
Albumin	3.6 g/dL	3.9-5.1 g/dL
Total bilirubin	3.2 mg/dL	0.2-1.2 mg/dL
Aspartate aminotransferase	17 U/L	11-30 U/L
Alanine aminotransferase	11 U/L	4-30 U/L
Lactase dehydrogenase	185 U/L	109-216 U/L
Creatine kinase	956 U/L	40-150 U/L
Blood urea nitrogen	18.8 mg/dL	8-20 mg/dL
Creatinine	0.68 mg/dL	0.63-1.03 mg/dL
Sodium	139 mEq/L	136-148 mEq/L
Potassium	2.7 mEq/L	3.6-5.0 mEq/L
Chloride	97 mEq/L	98-108 mEq/L
Glucose	181 mg/dL	70-109 mg/dL
Hemoglobin A1c	5.9%	5.6-5.9%

**Figure 2:**
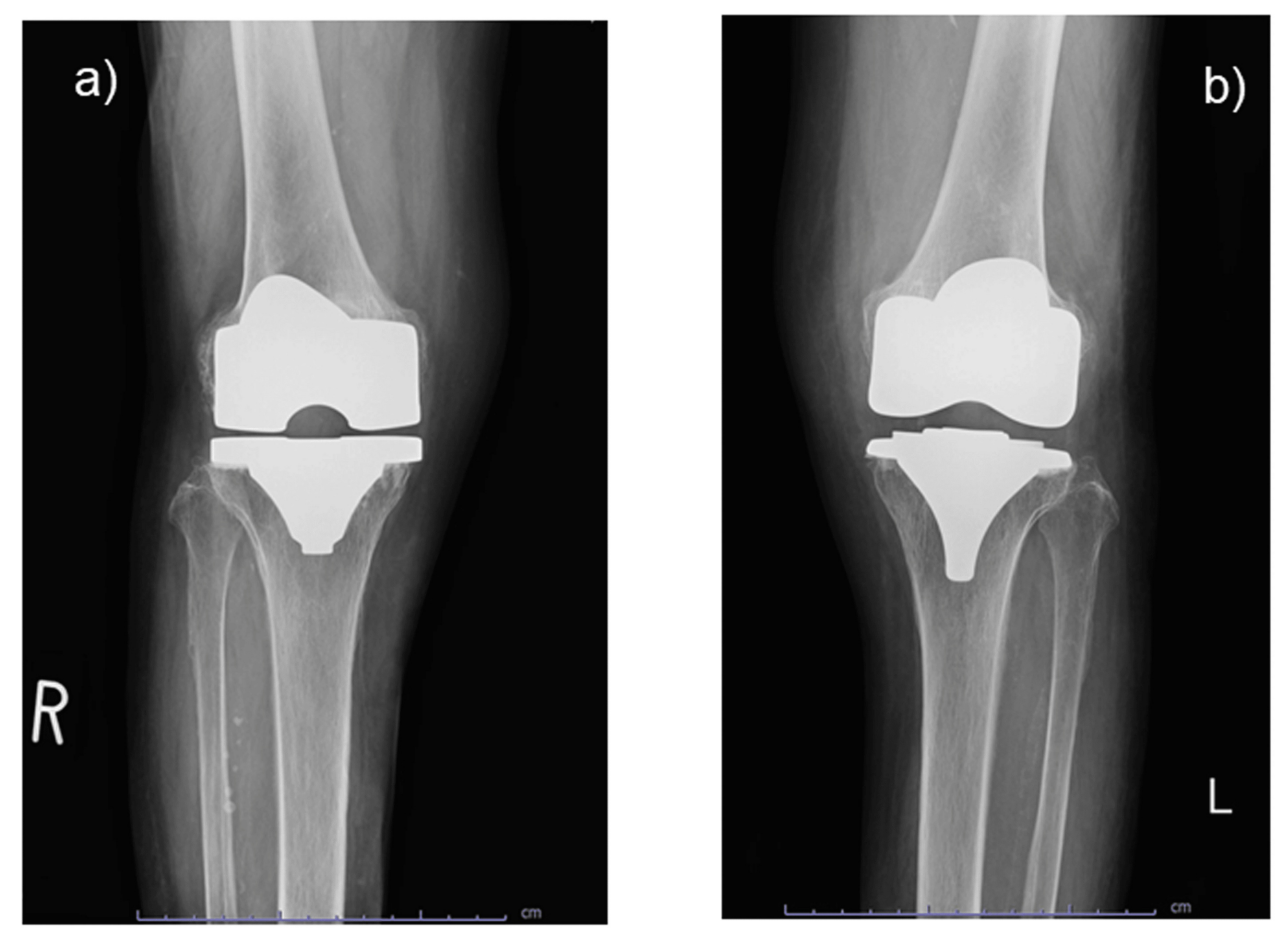
Radiography of both knees (a) Right knee. (b) Left knee. Knee radiography shows no fracture or prosthesis loosening.

The patient's white blood cell (WBC) count was 10,260/μL (neutrophils, 78.4%), and her C-reactive protein level was 15.82 mg/dL. Two sets of blood cultures yielded negative results. Knee radiography revealed no fractures or prosthetic loosening (Figure 2).

Synovial fluid analysis revealed yellow, viscous, non-turbid fluid with a WBC count of 30,950/μL, a glucose level of 91 mg/dL, positive CPP crystals, and negative Gram staining.

Based on the identification of CPP crystals in the synovial fluid, the patient was diagnosed with acute CPP crystal arthritis. Although septic arthritis was an important differential diagnosis given the history of TKA, negative Gram staining and culture results supported the diagnosis of acute CPP crystal arthritis.

Owing to the pain and difficulty walking, the patient was admitted. Celecoxib (200 mg/day) was initiated, resulting in rapid symptom improvement. Inflammatory marker levels decreased, peaked on day three, and normalized by day 17. The patient was discharged after 21 days of rehabilitation. No recurrence was observed during one year of follow-up.

## Discussion

Acute CPP crystal arthritis after TKA is exceedingly rare [6-8] but clinically important because its presentation closely mimics that of septic arthritis of the prosthetic joint. The novelty of this case lies in the development of acute CPP crystal arthritis more than 10 years after TKA. Additionally, this case underscores the importance of considering acute CPP crystal arthritis in the differential diagnosis of acute postoperative knee inflammation, since misdiagnosis may lead to unnecessary antibiotic therapy or surgical intervention.

The rarity of acute CPP crystal arthritis after TKA has been attributed to the absence of articular cartilage [1], which is typically required for CPP crystal deposition. However, previous reports have suggested that residual synovial tissue may undergo chondrogenic metaplasia and subsequently produce CPP crystals, providing a plausible mechanism for crystal-induced arthritis, even in prosthetic joints [3]. This mechanism is consistent with our findings, as CPP crystals were clearly identified in the synovial fluid. Chondrogenic metaplasia of residual synovial tissue after TKA may lead to the production of CPP crystals, and crystal‑induced arthritis has therefore been reported even in prosthetic joints. However, previously reported cases have generally occurred relatively early after surgery [4,5]. In contrast, the present case developed acute CPP crystal arthritis more than 10 years after TKA, representing a rare example with an exceptionally long interval.

Clinically, distinguishing acute CPP crystal arthritis from septic arthritis is challenging because both conditions present with fever, erythema, swelling, and severe joint pain. Septic arthritis of a prosthetic joint is a medical emergency that requires prompt antibiotic therapy and often surgical debridement or prosthesis revision [9]. Therefore, accurate diagnosis is essential. Synovial fluid analysis remains the cornerstone of differentiation. Although increased synovial WBC counts may be observed in both conditions, identification of CPP crystals, combined with negative Gram staining and culture results, strongly supports a diagnosis of acute CPP crystal arthritis.

The positive likelihood ratios for pyogenic arthritis based on joint aspirate WBC counts have been reported as follows: <25,000/μL: likelihood ratio of 0.32; ≥25,000/μL: likelihood ratio of 2.9; >50,000/μL: likelihood ratio of 7.7; and >100,000/μL: likelihood ratio of 28.0 [10]. As emphasized in the literature, joint aspiration should be performed before initiating antimicrobial therapy whenever possible to avoid diagnostic uncertainty and unnecessary treatment [9].

This case highlights the importance of including acute CPP crystal arthritis in the differential diagnosis of acute knee inflammation after TKA. Although uncommon, failure to recognize this condition may lead to overtreatment. Careful synovial fluid evaluation, including crystal analysis, cell count, Gram staining, and culture, allows clinicians to avoid unnecessary antibiotic use and surgery. Clinicians should be aware that acute CPP crystal and septic arthritis rarely coexist, underscoring the need for continued clinical vigilance and follow-up.

## Conclusions

This case highlights an uncommon presentation of acute CPP crystal arthritis occurring many years after TKA. Although CPP crystal arthritis is generally considered unlikely in prosthetic joints due to the absence of articular cartilage, our findings demonstrate that crystal‑induced inflammation can still develop long after surgery, likely through mechanisms such as chondrogenic metaplasia of residual synovial tissue. Clinicians should therefore recognize that acute CPP crystal arthritis may arise even more than a decade after TKA and should not exclude this diagnosis solely based on the time elapsed since arthroplasty.

Accurate differentiation between acute CPP crystal arthritis and prosthetic joint infection is essential, as the two conditions share similar clinical features but require fundamentally different management strategies. Prompt joint aspiration and comprehensive synovial fluid analysis - including crystal identification, cell count, Gram staining, and culture - are critical to avoid unnecessary antibiotic therapy or surgical intervention. Continued clinical vigilance and appropriate follow‑up are warranted to prevent misdiagnosis and reduce the risk of overtreatment and associated morbidity.
